# Identification and evaluation *in-vitro* of conserved peptides with high affinity to MHC-I as potential protective epitopes for Newcastle disease virus vaccines

**DOI:** 10.1186/s12917-023-03726-w

**Published:** 2023-10-07

**Authors:** Luis Tataje-Lavanda, Edith Málaga, Manuela Verastegui, Egma Mayta Huatuco, Eliana Icochea, Manolo Fernández-Díaz, Mirko Zimic

**Affiliations:** 1 Research and Development Laboratories, FARVET SAC, Chincha Alta, Ica, Peru; 2 Laboratory of Clinical Molecular Virology, Faculty of Biological Sciences, National University of San Marcos, Lima, Peru; 3 School of Human Medicine, Private University San Juan Bautista, Lima, Peru; 4 Research Laboratory On Infectious Diseases, Cayetano Heredia Peruvian University, Lima, Peru; 5 Avian Pathology Laboratory, Faculty of Veterinary Medicine, National University of San Marcos, Lima, Peru; 6 Bioinformatics, Molecular Biology, and Technological Developments Laboratory, Faculty of Science and Philosophy, Cayetano Heredia Peruvian University, Lima, Peru

**Keywords:** Epitope, Fusion, Hemagglutinin-neuraminidase, Newcastle disease virus, Peptide-based vaccine, Polymerase

## Abstract

**Background:**

Newcastle disease (ND) is a major threat to the poultry industry, leading to significant economic losses. The current ND vaccines, usually based on active or attenuated strains, are only partially effective and can cause adverse effects post-vaccination. Therefore, the development of safer and more efficient vaccines is necessary. Epitopes represent the antigenic portion of the pathogen and their identification and use for immunization could lead to safer and more effective vaccines. However, the prediction of protective epitopes for a pathogen is a major challenge, especially taking into account the immune system of the target species.

**Results:**

In this study, we utilized an artificial intelligence algorithm to predict ND virus (NDV) peptides that exhibit high affinity to the chicken MHC-I complex. We selected the peptides that are conserved across different NDV genotypes and absent in the chicken proteome. From the filtered peptides, we synthesized the five peptides with the highest affinities for the L, HN, and F proteins of NDV. We evaluated these peptides in-vitro for their ability to elicit cell-mediated immunity, which was measured by the lymphocyte proliferation in spleen cells of chickens previously immunized with NDV.

**Conclusions:**

Our study identified five peptides with high affinity to MHC-I that have the potential to serve as protective epitopes and could be utilized for the development of multi-epitope NDV vaccines. This approach can provide a safer and more efficient method for NDV immunization.

## Background

Newcastle disease (ND) is a viral infection that affects various systems of birds, including the neurological, gastrointestinal, reproductive, and respiratory systems. Clinical signs of ND include snoring, difficulty breathing, watery discharge from the nostrils, facial swelling, paralysis, tremors, and neck twisting, which is a sign of central nervous system involvement. ND is caused by paramyxovirus serotype 1 (APMV-1), also known as Newcastle disease virus (NDV), which has a genome comprising 15Kb of negative-sense single-stranded RNA (-ssRNA). NDV is highly contagious and can infect more than 200 bird species, significantly affecting poultry production worldwide [1–4].

ND is typically controlled using inactivated, live, or attenuated vaccines of genotypes I and II, including lentogenic, Hitchenner-B1-LaSota, V4, NDW, I2, and F strains. Inactivated vaccines are inoculated subcutaneously or intramuscularly, inducing a high level of humoral response with no side effects, although they induce a weak cellular response. In contrast, live and attenuated vaccines are typically inoculated via the ocular-nasal route, stimulating a mucosal immune response. Some genotypes, such as genotype II-LaSota, can induce a strong immune response without reverting to virulence or inducing recombination, although in some cases, it can be reactive in young birds, causing post-vaccination respiratory reactions [3, 5, 6].

Currently, there is growing interest in developing vaccines based on immunogenic subunits of the pathogen (epitopes), which are theoretically safer and potentially more effective. Epitopes are peptides that can bind to major histocompatibility complex type I (MHC-I) or type II (MHC-II) and be recognized by CD8/CD4 cells via the T-cell receptor (TCR), inducing a cellular (MHC-I) or humoral (MHC-II) response [7–9]. The prediction of epitopes from a pathogen genome analysis is desired for the development of multi-epitope vaccines [10, 11]. Bioinformatic design of an epitope vaccine involves analyzing the genome/proteome of a pathogen using immunoinformatics tools. While tools for estimating the affinity of peptides for MHC-I or MHC-II are currently reliable [12–14], those predicting the recognition of the MHC-I/peptide complex by the TCR are not yet so reliable [15]. Therefore, the ability of a peptide to induce a cellular response is usually assessed in *ex-vivo* assays where the ability of a peptide to evoke lymphocyte stimulation is measured [12, 13].

The development and use of multi-epitope vaccines against diseases in the poultry industry are currently limited [16–22], and most T-epitope vaccine designs target human diseases [23, 24]. This study aimed to select and evaluate conserved linear peptides of NDV in-silico and in-vitro with high affinity to chicken MHC-I and with the capacity to induce lymphocyte proliferation, potentially constituting epitopes with protective capacity against NDV. These epitopes could be used as multi-epitope vaccines.

## Results

### Prediction and selection of candidate MHC-I cognate epitopes

Out of the candidate epitopes that passed the filters, we selected five epitopes with the highest affinity score for MHC-I. Two of these epitopes are part of the large polymerase protein (L), two are from the haemagglutinin-neuraminidase (HN), and one is from the fusion protein (F) (Table 1).

**Table 1:** Evaluation of the cellular immune response to the 5 selected peptides by ELISpot assay

Antigen in the well of ELISpot plate	Peptide sequence (length)	Name of NDV protein	Spot Counting (Mean ± SD)		Statistical tests 95%		
			**Control Group**	**Vaccinated group:**	**Non-parametric method**	**Parametric methods**	
			**Spleen cells of non-immunized chickens**	**Spleen cells of immunized chickens**	**Kruskal–Wallis**	**Poisson regression**	**Negative binomial regression**
Peptide 01	FEELIHVNH (9 mers)	L	8.67 ± 5.92	133.67 ± 100.09	0.0065^a^	0.000^a^	0.000^a^
Peptide 02	FEDWVANY (8 mers)	HN	65.33 ± 114.81	209.83 ± 97.78	0.0250^a^	0.000^a^	0.044^a^
Peptide 03	KESVAATNE (9 mers)	F	9.33 ± 10.13	159.5 ± 141.86	0.0039^a^	0.000^a^	0.000^a^
Peptide 04	NEEREAKN (8 mers)	HN	42.33 ± 72.10	90.83 ± 83.23	0.1282	0.000^a^	0.241
Peptide 05	KEMIHVNH (8 mers)	L	8.17 ± 3.43	147.5 ± 114.61	0.0039^a^	0.000^a^	0.000^a^
Pool of peptides	Mix of 5 peptides^b^	L + HN + F	9.33 ± 5.79	191.67 ± 157.12	0.0039^a^	0.000^a^	0.000^a^
ConA	Positive control		667.33 ± 32.15	798.5 ± 117.17	0.0547	0.000^a^	0.002^a^
Cells with culture medium	Negative control		3.17 ± 4.02	40.33 ± 39.36	0.0202^a^	0.000^a^	0.000^a^

^a^Significant difference between vaccinated and control groups, ^b^Mix of 5 peptides in same concentration

*NDV* Newcastle Disease Virus, *ELISpot* Enzyme-linked immunospot, *ConA* Concavalin A, *L* Large polymerase, *HN* Hemagglutinin-neuraminidase, *F* Fusion

In Table 2, the amino acid differences of the 5 peptides tested against genotypes II (live vaccines) and XII (highly virulent) genomes are shown in red [25]. Sequence identity varies between 88 and 89%. The differences between the genomes evaluated and the designed peptides are highlighted in bold. Variations of genotype XII are shown as FMELIHVNH* (China, MT301958.1) and NEERESKN** (Peru, JN800306.2, KR732614.1).

**Table 2:**
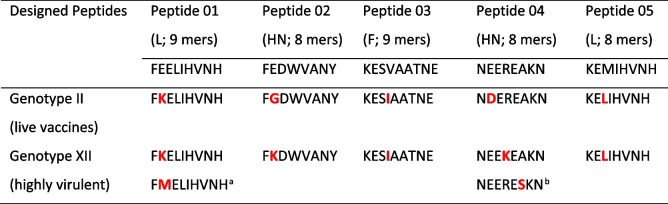
Comparison of designed peptides for various NDV genotypes with genotypes II (LaSota) and XII

Variations of genotype XII: ^a^China (MT301958.1), ^b^Peru (JN800306.2, KR732614.1). Amino acid variations in the 5 tested peptides against genotypes II (live vaccines) and XII (highly virulent) genomes are highlighted in red

### Measurement of the cellular immune response

**Fig. 1:**
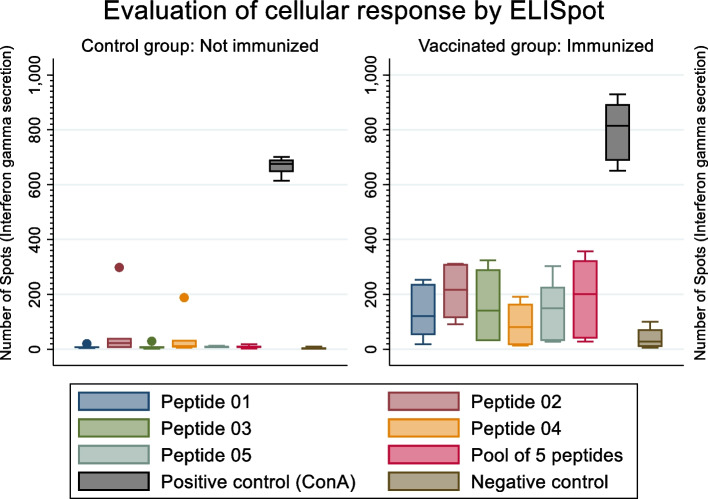
Evaluation of cellular response using IFN-γ-specific spot-forming cells. The box-and-whisker plot displays the results of the ELISpot assay when splenocytes from the Control Group (not immunized) and Vaccinated Group (previously immunized with NDV) were stimulated with different NDV peptides. The Y-axis represents the number of interferon gamma-specific spot-forming cells. Boxes represent the interquartile range (25th to 75th percentile), the median is indicated by a horizontal black line, and the whiskers extend to the most extreme data point. The positive control was splenocytes stimulated with Concanavalin A (ConA) in culture medium, while the negative control was splenocytes cultured in medium without any stimulus. The statistical analyses can be found in Table 1

The *ex-vivo* assay demonstrated significant differences in IFN-γ production between the groups tested with different peptides. The vaccinated group showed a higher number of spots than the control group. Additionally, in the vaccinated group, the samples stimulated with the epitopes exhibited a smaller spread in the number of spots than the control group. The number of INF-γ spots of epitopes 02 and 05 and the pool showed a negative asymmetric distribution, mostly clustering in the second quartile. The number of spots of epitopes 01, 03, and 04 showed a positive asymmetric distribution in the third quartile. Kruskal–Wallis, Poisson, and negative binomial tests confirmed significant differences in the number of spots between the vaccinated and control groups for epitopes 01, 02, 03, and 05. Epitope 04 was only significant with the Poisson test. The pool of the five peptides showed significant differences in all three tests (Fig. 1 and Table 1). In the ELISpot assay, both the positive control (Concanavalin A) and the negative controls (splenocytes in culture medium without stimulus) were used.

## Discussion

The ex-vivo assay showed higher secretion of INF-γ in the vaccinated group than in the control group for four out of the five epitopes studied. This indicates that the cytokine was produced by splenocytes with cellular memory when stimulated with the epitopes. Only one epitope did not show a significant difference from the control.

We performed a Poisson regression in which all p-values were significant. However, as a precaution, we also used negative binomial regression, which is more robust because it does not assume that the median coincides with the mean.

Reports on in-silico design of vaccines based on immunogenic epitopes for poultry are scarce. Among them are epitopes against viruses such as Fowl adenovirus serotype 4 (FAdV-4) [16], Infectious laryngotracheitis (ILTV) [17], and Infectious Bronchitis Bronchitis Virus (IBV) [18, 19]. In the case of NDV, we found three publications with selected epitopes of the haemagglutinin protein (HN) [20] and of the fusion protein (F) [21, 22, 26] to evoke the cellular response. These epitopes have 9 mers, and in some cases, were designed by methods involving artificial neural networks [21, 22, 26].

In the present study, the selection of 8- and 9-mer epitopes was aimed at evoking a cell-mediated immune response by T lymphocytes. Therefore, the selected epitopes were not only limited to NDV surface proteins (F and HN) but also included non-exposed proteins [20–22, 26]. Additionally, we opted to screen all available full and partial NDV protein sequences in GenBank, without focusing on a single strain type or genotype or geographic region. In theory, this approach would provide highly conserved and robust epitopes against possible NDV variants not considered in this study.

Generally, freely available tools are used to predict avian T-type viral epitopes, including NDV [16–19] viral epitopes, and in particular NDV [20–22, 26]. Using tools such as MCH-I Binding Predictions v2.24 available on the Immune Epitope Database and Analysis Resource (IEDB) website [27]. To use this tool, one must select the appropriate methodology (Consensus, Artificial Neural Network, Stabilized Matrix Method), choose pre-configured MHC-I alleles or upload an allele. The aforementioned authors used human alleles with high similarity to avian homologues since they did not have *Gallus gallus* alleles. In our study, we developed an *ad-hoc* tool based on artificial intelligence (AI), specifically an artificial neural network. We trained the AI with experimental data on the affinity of the majority MHC-I allele of the Cobb-500 chicken and a diverse set of peptides (data not shown). We refined the peptide selection by conservation and tolerance filtering of the epitopes, where conservation filtering aimed to find peptides present in as many NDV sequences as possible and tolerance filtering aimed to exclude peptides with potential autoimmune responses.

The importance of peptide affinity for the MHC complex as an epitope should not be overestimated, as it may not necessarily be recognized by the TCR receptor of CD8 T cells [10, 13, 28, 29]. Bioinformatics methods for predicting MHC-I/peptide complex recognition by the TCR receptor of T-CD8 lymphocytes and subsequent activation of the respective lymphocyte and stimulation of a cellular response are not yet completely reliable. Therefore, in this study, we preferred to experimentally assess the ability of peptides to stimulate lymphocyte memory in animals previously vaccinated with NDV using the ELISpot assay. A positive ELISpot result indicates that the peptides were bound to MHC-I during immunization and were recognized by the TCR receptor, inducing a cellular response and memory [30, 31].

*In-silico* peptides have been designed and evaluated in Sudan, Iran, and Uganda [20–22, 26] using various bioinformatics programs, but none of them followed up with *wet lab* evaluations. While in-silico predictions are improving over time, they cannot replace ex-vivo or in-vitro experimentation. Wet lab experiments are necessary to have a closer reference to reality. In our study, we limited ourselves to evaluating only the top 5 epitopes in a small number of SPF chicken spleens. Further in-vitro and in-vivo evaluations are still required before applying the peptides in farms. However, this is a first step towards turning our epitopes into NDV vaccines.

Our results suggest that the tested epitopes could serve as a booster vaccine against virulent variants, such as genotype XII (Table 2), as they stimulate cellular response. Alternatively, they could be used in combination with inactivated vaccines to avoid side effects. Such vaccines have several advantages, including large-scale production while keeping costs low, high reproducibility, favorable storage, and flexible distribution [29, 32]. The multi-epitope antigen could be delivered in several ways, such as a recombinant protein (tandem array of epitopes on a multi-epitope chain), within a viral vector, within a bacterial vector, in an encapsidated mRNA molecule, or in a lipid nanoparticle.

These findings motivate us to design new immunogenic epitopes against other pathogenic viruses of poultry interest. In the future, we will evaluate their use in synthetic multi-epitope proteins, which would protect against a variety of viral pathogens with the same vaccine. This study provides the first *ex-vivo* evidence that MHC-I-like T-type epitopes against NDV can evoke cellular responses. The immunoinformatics tools employed are suitable for the design of immunogenic T-type epitopes. Before this work, our team developed an artificial intelligence algorithm based on artificial neural networks to predict the affinity of an arbitrary peptide for the most frequent MHC-I allele of Cobb-500 chicken farms in Peru. The convolutional neural network was trained with experimental MHC-I/peptide affinity data (to be published).

### Conclusion

In conclusion, our study demonstrates the feasibility of using a combination of bioinformatics and experimental approaches to identify and evaluate potential vaccine candidates against NDV. The selected epitopes showed the ability to evoke a cell-mediated immune response in vaccinated animals, which is essential for protecting against NDV infection. Further studies are needed to evaluate the efficacy of these epitopes as vaccine candidates in field trials.

## Methods

**Fig. 2:**
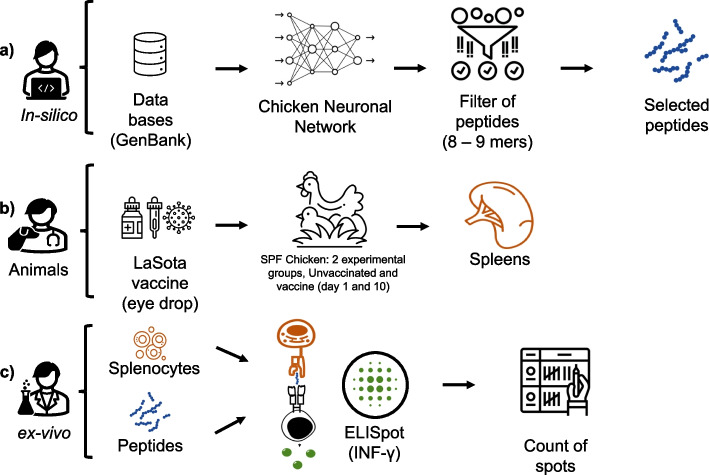
Flowchart of the selection procedure of candidate protective epitopes. **a** Design of immunogenic peptides: Mapping, prediction, and selection of linear type I class I epitopes of NDV with potential for protection. **b** Broiler management: Rearing, vaccination, and spleen removal from broilers. **c** Assessment of cellular response using ELISpot and spot counting: Higher numbers of spots indicate a greater immunogenic capacity of the peptide

The design and evaluation of MHC-I cognate NDV epitopes involved the use of bioinformatics tools, animal handling, and ex-vivo evaluation (Fig. 2).

### In-silico: Predicting candidate epitopes

We downloaded 21,374 genomic (partial and total) and protein sequences of NDV from various geographic regions, strains, and genotypes from GenBank as of the 2017 publication. The multifastsat.py v.1.4.9 script [33] was used to trim all sequences into segments overlapping by one amino acid (8 and 9 mers). An *ad-hoc* artificial neural network developed by our group (to be published) was utilized to predict MHC-I cognate epitopes. The epitopes were selected based on their highest affinity score and then filtered for conservation using tblastx (BLOSUM90, highest number of hits in GenBank) and tolerance against the *Gallus gallus* proteome (UniProt: UP000000539). To evaluate the conservation of the top five peptides, we performed a BLAST search (tblastn, word size: 2; BLOSUM90) against complete genomes of genotypes II (live vaccines) and XII (highly virulent). Finally, the selected epitopes were synthesized at a concentration of 10–14 mg and with a purity of greater than 75% by GenScript (USA).

### Animals: Biological samples

The study involved two groups of hens (2 vaccinated and 2 control) obtained from the hatchery of the FARVET SAC company in (Ica, Peru). The birds were specific pathogen-free (SPF) Hyline female chicks and were reared in the Poultry Pathology Laboratory of the Universidad Nacional Mayor de San Marcos (UNMSM). The handling of the birds followed protocols approved by the Animal Welfare Ethics Committee (CEBA) of the UNMSM. The birds were housed in cages at the experimental facility, where conditions were carefully controlled, maintaining temperatures between 20°C to 22°C and a 12-h light–dark cycle. They had ad libitum access to food (crushed corn) and water throughout the study period. The vaccinated group received NDV LaSota vaccine (≥ 10^8.0^ TCID 50%) on days 1 and 10. The vaccine was diluted in 30 ml of distilled water and administered by eye drop in doses of 0.05 ml per bird. At week 8 (with an average weight of 595g), birds in both groups were sacrificed by cervical dislocation, and spleens were collected in DMEM medium (Gibco, USA) with antibiotics and transported cold.

### *Ex-vivo*: Measurement of cell-mediated immune response in spleen mononuclear cells

Splenocytes were perfused in DMEM medium (Gibco, USA) supplemented with 10% fetal bovine serum (HyClone, USA), filtered at 70 µm and washed at 300 × g for 10 min. Lymphocytes were obtained by gradient centrifugation with Histopaque 1077 (Sigma, USA). The cells were then assessed for viability and concentrated to 5 × 10^6^ cells/ml.

The cellular response was measured by ELISpot assay [34, 35] in triplicate using the Ch. IFN-γ Cytoset kit (Thermo Fisher, USA). To each well, 50 µl of culture medium, 50 µl of cells (800,000 cells), and 50 µl of antigen (10 µg/epitope well) were added. As a positive control, 5 µg/ml concanavalin A (ConA) was used, while the negative control consisted of cells with culture medium only. The plate was then incubated at 41 °C with 5% CO_2_ for 48 h. INF-γ secretion was detected with 0.5 µg/ml of biotin-conjugated anti-chIFN-γ and 100 µl of Streptavidin-HRP. The plate was read with the AID EliSpot Reader System ELR04 (AID, Germany), and spots were counted manually using ImageJ v.1.53k software [36]. The difference in the number of spots between groups was assessed by Kruskal–Wallis, Poisson, and negative binomial tests using STATA v.14.1.

## Data Availability

Additional data are available from the authors upon reasonable request, and with permission of FARVET SAC company.

## Abbreviations

AI: Artificial intelligence
APMV-1: Avian paramyxovirus serotype 1
ConA: Concavalin A
F: Fusion
FAdV-4: Fowl adenovirus serotype 4
HN: Hemagglutinin-neuraminidase
IBV: Infectious bronchitis bronchitis virus
ILT: Infectious laryngotracheitis
L: Large polymerase
MHC-I: Major histocompatibility complex type I
MHC-II: Major histocompatibility complex type II
mRNA: Messenger RNA
ND: Newcastle disease
NDV: Newcastle disease virus
-ssRNA: Negative-sense single-stranded RNA
TCR: T-cell receptor
